# Supplementary material for: Evidence supported by Mendelian randomization: impact on inflammatory factors in knee osteoarthritis
Source: Front Med (Lausanne). 2024 May 28;11:1382836. doi: 10.3389/fmed.2024.1382836 (PMC11165061; doi:10.3389/fmed.2024.1382836)
Supplement: Supplementary file 1 [file Data_Sheet_1.ZIP › data/GCST90274778_5e6/778.forest.pdf]

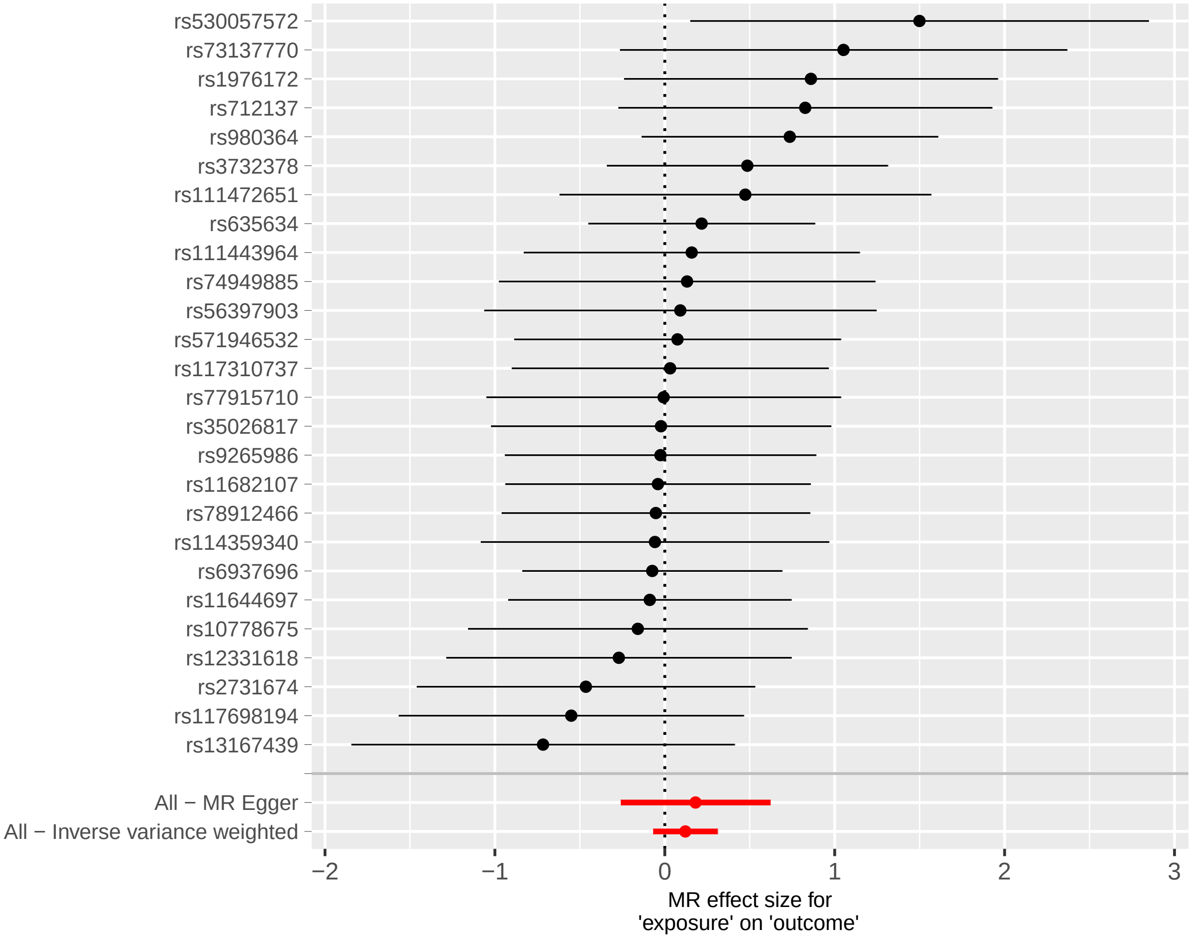
rs530057572
rs73137770
rs1976172
rs712137
rs980364
rs3732378
rs111472651
rs635634
rs111443964
rs74949885
rs56397903
rs571946532
rs117310737
rs77915710
rs35026817
rs9265986
rs11682107
rs78912466
rs114359340
rs6937696
rs11644697
rs10778675
rs12331618
rs2731674
rs117698194
rs13167439
All − MR Egger
All − Inverse variance weighted
−2
−1
0
1
2
3
MR effect size for
'exposure' on 'outcome'
